# The inverted U-shaped association between blood fibrinogen and rehospitalization risk in patients with heart failure

**DOI:** 10.1038/s41598-024-66002-3

**Published:** 2024-07-01

**Authors:** Zhenyan Xu, Hualong Liu, Meilan Zhu, Ying Huang

**Affiliations:** 1 Rehabilitation Department, The Second Affiliated Hospital, Jiangxi Medical College, Nanchang University, Nanchang City, 330006 Jiangxi China; 2 Cardiovascular Department, The Second Affiliated Hospital, Jiangxi Medical College, Nanchang University, Nanchang City, 330006 Jiangxi China

**Keywords:** Fibrinogen, Heart failure, Readmission risk, Death risk, Chinese population, Biochemistry, Biogeochemistry, Biomarkers

## Abstract

Fibrinogen, a biomarker of thrombosis and inflammation, is related to a high risk for cardiovascular diseases. However, studies on the prognostic value of blood fibrinogen concentrations for heart failure (HF) patients are few and controversial. We performed a retrospective analysis among acute or deteriorating chronic HF patients admitted to a hospital in Sichuan, China, between 2016 and 2019, integrating electronic health care records and external outcome data (N = 1532). During 6 months of follow-up, 579 HF patients were readmitted within 6 months, and 46 of them died. Surprisingly, we found an inverted U-shaped association of blood fibrinogen levels with risk of readmission within 6 months but not with risk of death within 6 months. It was found that HF patients had the highest risk for readmission within 6 months after reaching the turning point for blood fibrinogen (2.4 g/L). In HF patients with low fibrinogen levels < 2.4 g/L, elevated fibrinogen concentrations were still significantly associated with a higher risk for readmission within 6 months [OR = 2.3, 95% CI (1.2, 4.6); *P* = 0.014] after controlling for relevant covariates. There was no significant association between blood fibrinogen and readmission within 6 months [(OR = 1.0, 95% CI (0.9, 1.1); *P* = 0.675] in HF patients with high fibrinogen (> 2.4 g/L). The effect difference for the two subgroups was significant (*P* = 0.014). However, we did not observe any association between blood fibrinogen and death within 6 months stratified by the turning point, and the effect difference for the stratification was not significant (*P* = 0.380). We observed an inverted U-shaped association between blood fibrinogen and rehospitalization risk in HF patients for the first time. Additionally, our results did not support that elevated blood fibrinogen was related to increased death risk after discharge.

## Introduction

Heart failure (HF) is a comprehensive manifestation of deteriorating cardiac function and is considered an important cause of death among common cardiovascular diseases (CVDs)^1–3^. Although significant improvements in anti-HF drugs and strategies have been made in recent years, the prognosis for HF patients remains poor^3^. Authoritative evidence showed that the one-year survival rate in the HF population was 74.2% in 2000, while the survival rate was only 80.8% in 2016^4^. Moreover, research has noted that the rehospitalization rate within 2–3 months after discharge for HF-hospitalized patients reached 30%^5^. The current poor situation encourages researchers to further explore the pathological mechanisms and accurate risk stratification for HF to better help clinicians perform appropriate medical decision-making and improve the prognosis in these patients.

Fibrinogen, as a 340 kDa glycoprotein, plays a key role in multiple pathological processes^6^. Numerous clinical and basic studies have demonstrated that elevated plasma concentrations of fibrinogen are a risk factor for developing CVDs^7–11^. For example, a prospective observational study including 6140 patients with coronary artery disease (CAD) undergoing percutaneous coronary intervention (PCI) reported that elevated blood fibrinogen concentrations were significantly associated with long-term all-cause mortality (HR = 1.86; 95% CI 1.28–2.69) and cardiac mortality (HR = 1.82; 95% CI 1.15–2.89)^8^. A cross-sectional study including 1096 patients with type 2 diabetes mellitus revealed that blood fibrinogen concentrations were an independent risk factor for peripheral arterial disease (PAD)^9^. In an observational study involving 153 patients categorized into two groups (patients with acute ischemic stroke and patients with risk factors but no stroke), they found that patients with ischemic stroke had a significantly increased mean plasma fibrinogen concentration (> 4 g/L)^10^. A significant association between blood fibrinogen concentrations and the presence of ischemic lesions on cerebral computed tomography was observed: patients with a fibrinogen concentration > 3.41 g/L showed a 3.29-fold increased risk of ischemic lesions^10^. Moreover, a prospective study containing 14,916 men aged 40–84 years in the Physicians’ Health Study evaluated the relationship between the plasma concentration of fibrinogen and myocardial infarction (MI) risk^11^, reporting that a high blood fibrinogen concentration (≥ 343 mg/dL) had a twofold increase in MI risk (RR = 2.09; 95% CI 1.15–3.78) compared with those with fibrinogen below 343 mg/dL, independent of other CVD risk factors^10^. Moreover, a previous meta-analysis with 1773 men who were free of HF or cardiac arrhythmias reported 131 sudden cardiac deaths within 22 years of follow-up, thus suggesting that blood fibrinogen concentrations were positively, log-linearly and independently associated with an increased risk of sudden cardiac death^12^. However, there are currently few or unclear explorations for the association of blood fibrinogen concentration with prognosis in HF patients. Only two clinical studies have primarily evaluated the effect of blood fibrinogen on the prognosis of HF, and their results are controversial and have obvious limitations. One of those studies reported that high fibrinogen levels (≥ 284 mg/dL) independently predicted 90-day mortality in critically ill patients with acute exacerbation of chronic HF, but those authors emphasized that their findings need to be further validated by large prospective studies with longer follow-up times^13^. Another study also only analyzed 120 patients with chronic HF and did not observe that elevated fibrinogen levels were associated with a high risk for all-cause mortality during a follow-up period of 2 years^14^.

In this study, our objective was to further evaluate the value of blood fibrinogen for predicting prognosis (risk for readmission and all-cause mortality within 6 months) with a large sample size of elderly HF patients (N = 1532) who were admitted to a hospital in Sichuan, China between 2016 and 2019.

## Materials and methods

### HF patients

**Figure 1:**
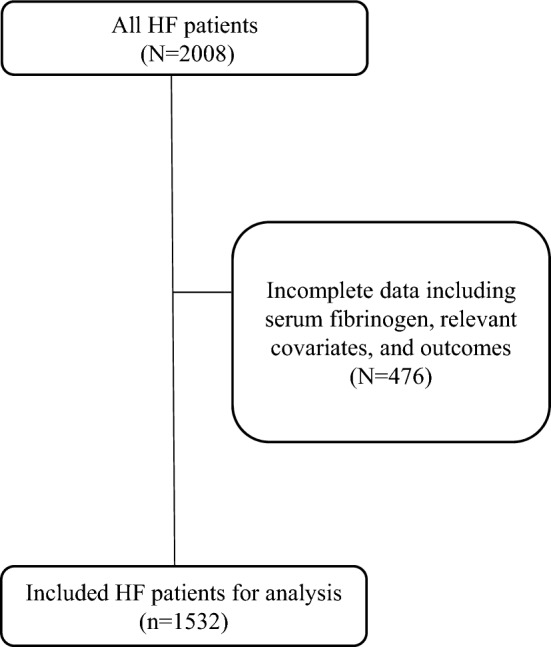
Flow chart of final HF patients for analysis.

Our study subjects comprised a representative sample from a large number of Chinese HF individuals (N = 2008) who had been admitted to Zigong Fourth People’s Hospital with HF between December 2016 and June 2019^15,16^. These patients were hospitalized due to acute or worsening chronic HF, and electronic health care records and external outcome data were integrated. Therefore, this study was designed as a cohort study to investigate risk factors for related outcomes (death risk and readmission risk)^15,16^. Of a total of 2008 HF patients selected into the dataset, subsequent hospital readmission and mortality risk were collected from a mandatory follow-up visit at 28 days, 3 months and 6 months^15,16^. The follow-up visit was replaced by a telephone if the HF patients were not able to visit the clinical center. According to the European Society of Cardiology (ESC) criteria^17^, HF was defined as follows: (1) the presence of symptoms and/or signs of HF; (2) elevated levels of BNPs (BNP > 35 pg/mL and/or NT-proBNP > 125 pg/mL); and (3) objective evidence of other cardiac functional and structural alterations underlying HF. In case of uncertainty, a stress test or invasively measuring elevated left ventricle (LV) filling pressure may have been needed to confirm the diagnosis^17^. For the present analyses, after excluding HF patients with missing information on blood fibrinogen, relevant covariates and outcomes (N = 476), a final cohort of 1532 individuals with nonmissing information was enrolled in our analysis. Figure 1 shows the process of HF patient inclusion. The research ethics committee of Zigong Fourth People’s Hospital (Approval Number: 2020–010) approved the study, and informed consent was waived by the research ethics committee of Zigong Fourth People’s Hospital due to the retrospective design that all methods were performed in accordance with the Declaration of Helsinki guidelines. We obtained variable information such as demographics, admission characteristics, laboratory indicators and medications. The author (Ying Huang) completed the required training course, and obtained the access to the database project and was responsible for data extraction (https://physionet.org/content/heart-failure-zigong/1.2/).

### Covariates

Clinical characteristics at baseline, including demographic characteristics [age, sex and body mass index (BMI)], admission characteristics (admission pathway and vital signs), composite diseases, type of HF, New York Heart Association (NYHA) cardiac function classification and laboratory measurements, were collected on the day of hospital admission^15,16^. The admission pathway included both emergency and non-emergency admissions. Vital signs included body temperature, pulse, respiration rate, systolic blood pressure (SBP), diastolic blood pressure (DBP) and mean arterial blood pressure (MBP). Composite diseases included congestive HF, MI, cerebrovascular disease, diabetes, peripheral vascular disease (PVD), moderate-to-severe chronic kidney disease (CKD), dementia, chronic obstructive pulmonary disease (COPD) and liver disease. HF type was mainly divided into left, right or both HF. NYHA cardiac function was mainly classified as II, III and IV. Laboratory measurements mainly included creatinine, glomerular filtration rate (mL/min/1.73 m^2^) and other routine tests upon admission. BMI was equal to weight (kg)/square of height (m). Moderate-to-severe CKD was defined as CKD with a glomerular filtration rate < 60 mL/min.

### Follow-up outcomes

This dataset was collected with the goal of developing a model to predict the emergency readmission of discharged HF patients^15,16^. For the present analyses, follow-up outcomes were defined as risk for readmission within 6 months and risk for death within 6 months.

### Statistical analysis

We used descriptive analyses to summarize the admission characteristics for these HF patients. First, cross-sectional associations of blood fibrinogen levels with readmission within 6 months and death within 6 months were evaluated by using calculated logistic regression (adjusted for age and sex in Model 1; adjusted for age, sex, BMI, MAP, type of heart failure and NYHA cardiac function classification in Model 2). All analyses were performed using odds ratios (ORs) with 95% confidence intervals (CIs). To further characterize the shapes of associations, a smooth curve was used to confirm real-world relationships between blood fibrinogen levels and readmission within 6 months and death within 6 months with ORs and 95% CIs. As the association showed a nonlinear shape, a turning point for blood fibrinogen (2.4 g/L) was detected in which HF patients had the highest risk for readmission within 6 months. Therefore, we conducted hierarchical analysis through the turning point (HF patients with fibrinogen < 2.4 g/L and with fibrinogen > 2.4 g/L). Finally, subgroup analyses were performed to examine statistical evidence of effect modification by clinically relevant characteristics, such as comorbidities including congestive HF, MI, cerebrovascular disease, diabetes, PVD, moderate-to-severe CKD, dementia, COPD and liver disease. Empower version 4.1 was used for analysis, and *P* ≤ 0.05 was considered to indicate statistical significance.

### Ethics approval

The research ethics committee of Zigong Fourth People’s Hospital (Approval Number: 2020–010) approved the study, and informed consent was waived by the research ethics committee of Zigong Fourth People’s Hospital due to the retrospective design that all methods were performed in accordance with the Declaration of Helsinki guidelines.

## Results

### Admission characteristics in patients with HF

The admission characteristics for a total of 1532 HF patients who met the inclusion criteria are described in Table 1. The number of male individuals among these HF patients was 637 (41.58%), and the number of individuals > 60 years old was 1401 (91.45%). The median blood fibrinogen level in all HF patients was 3.05 (2.51–3.76) g/L. Among these patients, the number for NYHA cardiac function classification from II to IV in these patients was 265 (17.30%), 800 (52.22%) and 467 (30.48%), respectively. Diabetes (23.56%) and moderate-to-severe CKD (23.43%) were the most common comorbidities. Because 1426 (93.08%) of them had congestive HF, 47.72% of HF patients were admitted to the emergency room with worsening HF.

**Table 1:** Admission characteristics in patients with HF (N = 1532).

Baseline variables	
Age (years)	
≥ 60, n (%)	1401 (91.45%)
< 60, n (%)	131 (8.55%)
Gender	
Male, n (%)	637 (41.58%)
Female, n (%)	895 (58.42%)
BMI (kg/m^2^)	20.76 (18.42–23.46)
Admission characteristics	
Non emergency, n (%)	801 (52.28%)
Emergency, n (%)	731 (47.72%)
Body temperature (°C)	36.30 (36.20–36.50)
Pulse (beats per minute)	82.50 (70.00–98.00)
Respiration (beats per minute)	19 (18–19)
SBP (mmHg)	130.00 (113.00–148.00)
DBP (mmHg)	76.00 (65.75–86.00)
MAP (mmHg)	94.00 (83.33–106.00)
Discharge day (days)	8.00 (6.00–10.00)
Composite diseases	
Myocardial infarction, n (%)	111 (7.25%)
Congestive heart failure, n (%)	1426 (93.08%)
Peripheral vascular disease, n (%)	77 (5.03%)
Cerebrovascular disease, n (%)	108 (7.05%)
Dementia, n (%)	92 (6.01%)
COPD, n (%)	178 (11.62%)
Diabetes, n (%)	361 (23.56%)
Moderate to severe CKD, n (%)	359 (23.43%)
Liver disease, n (%)	64 (4.18%)
Type of HF	
Left, n (%)	352 (22.98%)
Right, n (%)	45 (2.94%)
Both, n (%)	1135 (74.09%)
NYHA cardiac function classification	
II, n (%)	265 (17.30%)
III, n (%)	800 (52.22%)
IV, n (%)	467 (30.48%)
Laboratory measurement on admission	
D-dimer (mg/L)	1.22 (0.79–2.17)
Creatinine (umol/L)	87.60 (65.38–122.60)
Urea (mmol/L)	8.00 (5.86–11.50)
Glomerular filtration rate (mL/min/1.73 m^2^)	64.67 (41.38–89.18)
Red blood cell (10^12^/L)	3.90 (3.44–4.31)
Hemoglobin (g/L)	118.00 (102.00–132.00)
Platelet (10^9^/L)	136.00 (102.75–178.00)
International normalized ratio	1.21 (1.13–1.36)
Fibrinogen (g/L)	3.05 (2.51–3.76)
Brain natriuretic peptide (pg/mL)	765.58 (319.34–1784.06)
Albumin (g/L)	36.80 (33.50–39.90)
Cholesterol (mmol/L)	3.61 (2.98–4.33)
Low density lipoprotein cholesterol (mmol/L)	1.76 (1.32–2.29)
Triglyceride (mmol/L)	0.97 (0.71–1.31)
Follow-up outcomes	
Readmission within 6 months, n (%)	579 (37.79%)
Death within 6 months, n (%)	46 (3.00%)

*HF* heart failure, *BMI* body mass index, *SBP* systolic blood pressure, *DBP* diastolic blood pressure, *MAP* mean arterial pressure, *COPD* chronic obstructive pulmonary disease, *CKD* chronic kidney disease, *Moderate to severe CKD* chronic kidney disease with glomerular filtration rate < 60 mL/min, *NYHA* New York Heart Association.

Importantly, during 6 months of follow-up, 579 (37.79%) of these HF patients were readmitted within 6 months, and 46 (3.00%) died within 6 months. Then, we used logistic regression to explore the association of blood fibrinogen level with risk for readmission within 6 months and risk for death within 6 months in Table 2. Not surprisingly, we did not observe significant associations between blood fibrinogen and readmission within 6 months [OR = 1.03, 95% CI (0.93, 1.13); *P* = 0.6174] or death within 6 months [OR = 1.23, 95% CI (0.96, 1.57); *P* = 0.1065] in Model 1 when only covariates including age and sex were adjusted for. After other covariates, including BMI, MAP, type of heart failure, NYHA cardiac function classification and creatinine, were further added into the above model, their associations of blood fibrinogen with readmission within 6 months [OR = 1.04, 95% CI (0.94, 1.15); *P* = 0.4116] and death within 6 months [OR = 1.16, 95% CI (0.89, 1.51); *P* = 0.2713] remained meaningless.

**Table 2:** The association between blood fibrinogen and outcomes within 6 months in HF patients.

Outcomes	Model 1		Model 2	
	OR (95% CI)	*P* value	OR (95% CI)	*P* value
Readmission within 6 months	1.03 (0.93, 1.13)	0.6174	1.04 (0.94, 1.15)	0.4116
Death within 6 months	1.23 (0.96, 1.57)	0.1065	1.16 (0.89, 1.51)	0.2713

Model 1: Adjusted for age and gender.

Model 2: Adjusted for age, gender, BMI, MAP, type of heart failure, NYHA cardiac function classification and creatinine.

*HF* heart failure, *MAP* mean arterial pressure, *BMI* body mass index, *NYHA* New York Heart Association.

### Curvilinear association between blood fibrinogen and the outcomes within 6 months in HF patients

**Figure 2:**
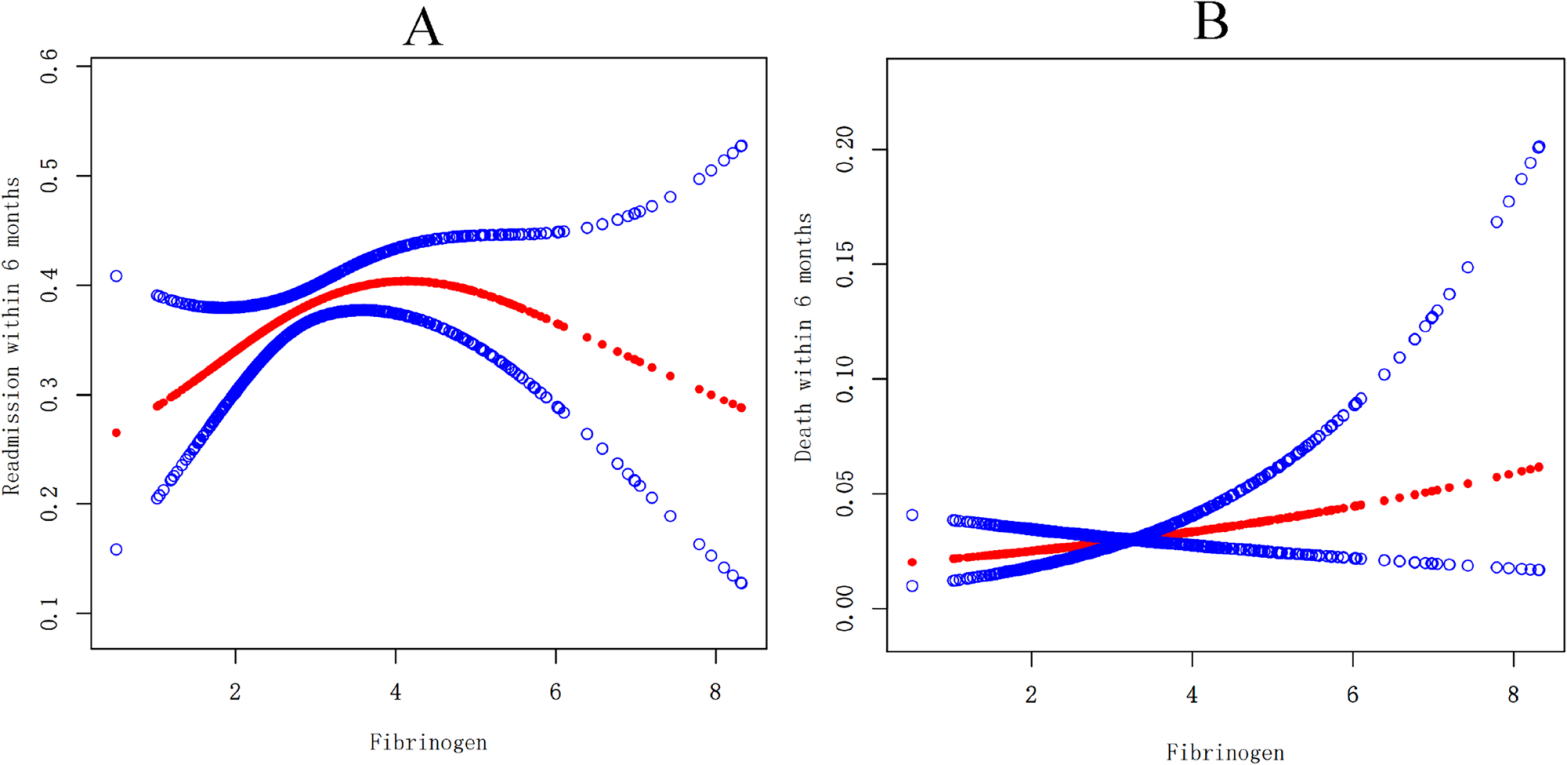
Curve analysis for associations between blood fibrinogen and risk for readmission and death within 6 months in HF patients.

**Figure 3:**
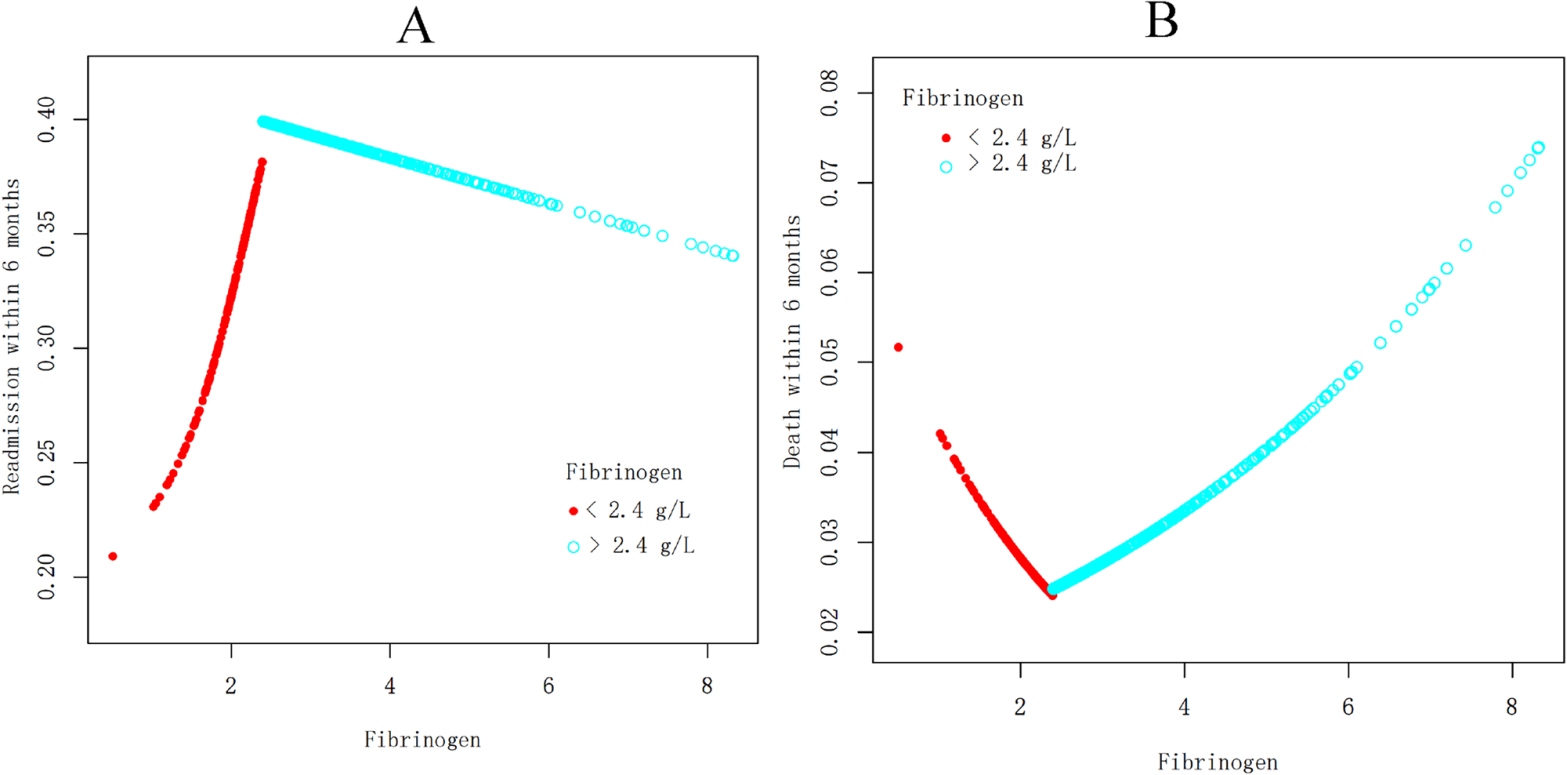
Stratified analysis for associations between blood fibrinogen and risk for readmission and death within 6 months in HF patients.

We further explored whether there was a curvilinear association between blood fibrinogen and adverse outcomes within 6 months (readmission and death). Unexpectedly, we observed an inverted U-shaped relationship between blood fibrinogen and risk for readmission within 6 months (Fig. 2A, P = 0.163) rather than death within 6 months (Fig. 2B, P = 0.272) after age, sex, BMI, MAP, type of heart failure, NYHA cardiac function classification and creatinine were adjusted. It was found that HF patients had the highest risk for readmission within 6 months after reaching the turning point for blood fibrinogen (2.4 g/L). This is an interesting and even strange discovery because the normal value of fibrinogen is 1.5–3.5 g/L. Therefore, grouping analysis through the turning point was performed (the low group with fibrinogen < 2.4 g/L and high group with fibrinogen > 2.4 g/L), as shown in Table 3. In HF patients with low fibrinogen < 2.4 g/L, we found that elevated fibrinogen concentrations of 1 g/L were still significantly associated with an increased risk of 130% for readmission within 6 months [OR = 2.3, 95% CI (1.2, 4.6); *P* = 0.014] after controlling for covariates including age, sex, BMI, MAP, type of heart failure, NYHA cardiac function classification and creatinine. Interestingly, there was no significant association between fibrinogen and readmission within 6 months [OR = 1.0, 95% CI (0.9, 1.1); *P* = 0.675] in HF patients with high fibrinogen > 2.4 g/L. The log likelihood ratio tests for the two subgroups was significant (*P* = 0.014). However, we still did not observe any significant associations between blood fibrinogen and death within 6 months stratified by a turning point for blood fibrinogen (2.4 g/L), and the effect difference for the stratification was not significant (*P* = 0.380). In addition, we also conducted stratified analysis by smooth curve fitting and found that the results were similar to the above (Fig. 3A and 3B).

**Table 3:** The turning point analysis for association between blood fibrinogen and outcomes within 6 months in HF patients.

Turning point for serum fibrinogen	Readmission within 6 months		Death within 6 months	
	OR (95% CI)	*P* value	OR (95% CI)	*P* value
Turning point	2.4 g/L		2.4 g/L	
< Turning point	2.3 (1.2, 4.6)	0.014	0.3 (0.0, 4.0)	0.390
> Turning point	1.0 (0.9, 1.1)	0.675	1.2 (0.9, 1.6)	0.180
Log Likelihood Ratio Tests	0.4 (0.2, 0.9)	0.014	3.6 (0.3, 46.6)	0.380

Adjusted for age, gender, BMI, MAP, type of heart failure, NYHA cardiac function classification and creatinine.

*HF* heart failure, *MAP* mean arterial pressure, *BMI* body mass index, *NYHA* New York Heart Association.

### Subgroup analysis of composite diseases for readmission risk within 6 months in HF patients

Subgroup analysis was conducted to evaluate the association between blood fibrinogen and risk for readmission and death within 6 months in different subgroups by composite diseases (Table 4). Significant differences in stratification for the association between fibrinogen and readmission within 6 months were not observed in MI, congestive heart failure, peripheral vascular disease, cerebrovascular disease, dementia, COPD, diabetes, moderate-to-severe CKD and liver disease respectively (all P > 0.05). Predictably, these subgroup analyses also showed no association between blood fibrinogen and death risk within 6 months (data not shown).

**Table 4:** The stratified analysis for association between blood fibrinogen and readmission within 6 months in HF patients.

Covariates	Readmission within 6 months	
	OR (95% CI)	*P* value
Myocardial infarction		
Yes (n = 111)	1.18 (0.81, 1.73)	0.3941
No (n = 1421)	1.03 (0.92, 1.14)	0.6397
Congestive heart failure		
Yes (n = 1426)	1.04 (0.94, 1.16)	0.4313
No (n = 106)	1.21 (0.76, 1.92)	0.4235
Peripheral vascular disease		
Yes (n = 77)	1.45 (0.65, 3.25)	0.3605
No (n = 1455)	1.05 (0.95, 1.16)	0.3784
Cerebrovascular disease		
Yes (n = 108)	1.57 (0.96, 2.57)	0.0725
No (n = 1424)	1.03 (0.93, 1.14)	0.5973
Dementia		
Yes (n = 92)	0.91 (0.63, 1.30)	0.5950
No (n = 1440)	1.06 (0.95, 1.17)	0.3206
COPD		
Yes (n = 178)	1.02 (0.75, 1.39)	0.8995
No (n = 1354)	1.04 (0.94, 1.16)	0.4423
Diabetes		
Yes (n = 361)	0.97 (0.79, 1.18)	0.7411
No (n = 1171)	1.06 (0.94, 1.19)	0.3771
Moderate to severe CKD		
Yes (n = 359)	1.09 (0.90, 1.32)	0.3719
No (n = 1173)	1.00 (0.89, 1.13)	0.9373
Liver disease		
Yes (n = 64)	2.36 (1.09, 5.15)	0.0304
No (n = 1468)	1.03 (0.93, 1.14)	0.5845

Adjusted for age, gender, BMI, MAP, type of heart failure, NYHA cardiac function classification and creatinine.

HF: heart failure; COPD: chronic obstructive pulmonary disease; CKD: chronic kidney disease; Moderate to severe CKD: chronic kidney disease with glomerular filtration rate < 60 ml/min; MAP: mean arterial pressure; NYHA: New York Heart Association.

## Discussion

Our analysis using an electronic health database comprising patients with HF from a hospital in Sichuan, China, between 2016 and 2019 provided several new findings that have not been reported previously. (1) Indeed, blood fibrinogen was not strongly correlated with the risk for death within 6 months in HF patients after controlling for some traditional and nontraditional risk factors. (2) We additionally found a nonlinear (inverted U-shaped) association of baseline fibrinogen with the risk for readmission within 6 months. It was found that HF patients had the highest risk for readmission within 6 months after reaching the turning point for blood fibrinogen (2.4 g/L).

A large amount of evidence has confirmed that increased blood fibrinogen levels contribute to CVD risk and worsen prognosis via platelet aggregation, plasma viscosity and fibrin formation in various populations. For instance, Danesh et al. conducted a meta-analysis of 31 prospective studies, indicating that each 1 g/L increase in blood fibrinogen concentration could contribute to an increased risk of 142% for CAD, 106% for stroke and 176% for other vascular deaths^18^. These significant relationships were independent of age, sex and other cardiovascular risk factors^18^. Another meta-analysis of 52 prospective studies performed by Kaptoge et al. involving 246,669 individuals without previously diagnosed CVD examined the prognostic effects of blood fibrinogen levels on CVD risk^19^. These researchers reported that elevated blood fibrinogen levels were related to an increased risk of 15% for CVD and drew a conclusion that assessing blood fibrinogen levels may be a very favorable indicator for detecting an intermediate CVD risk^19^. In addition, there is evidence also supporting that blood fibrinogen may be a valuable biomarker for subclinical atherosclerosis. In the Coronary Artery Risk Development in Young Adults study including 1396 participants aged 25–37 who were assessed for carotid intimal/medial thickness (CIMT) and coronary artery calcification (CAC)^20^, Green et al. found that the prevalence values of CAC with increasing quartiles of fibrinogen were 14.4%, 15.2%, 20.0% and 29.1%, and a similar trend was observed for CIMT. They concluded that increased fibrinogen concentration in these individuals was independently associated with subclinical CVDs^20^. Although these clinical investigations have populations with various CVDs, their conclusions were almost similar that high fibrinogen is closely related to poor prognosis, which is partly consistent with the results of our study. We found that HF patients with blood fibrinogen at the turning point (2.4 g/L) had the highest risk for readmission within 6 months, and elevated baseline fibrinogen independently increased the risk of readmission by 130% within 6 months in HF patients with fibrinogen < 2.4 g/L with each 1 g/L increase, while there was no significant association in these patients with high fibrinogen > 2.4 g/L. This is an interesting and even strange discovery because the normal value of fibrinogen in blood is 1.5–3.5 g/L, with a half-life of 3–5 days in generally healthy people^21–23^. In fact, many risk factors and health statuses could increase blood fibrinogen concentrations, including older age, female sex, Black race, smoking and drinking, hypertension, obesity, diabetes, metabolic syndrome and others. Elderly patients with HF are often accompanied by these risk factors, placing them in a hypercoagulable state and increasing their rehospitalization risk. Another potential explanation is that fibrinogen is also an acute phase protein, as its biosynthesis increases during the inflammatory process^24–26^. An increased systemic inflammatory response may contribute to a high risk for cardiovascular events requiring readmission, along with elevated blood fibrinogen concentrations (< 2.4 g/L).

In fact, few studies have reported the prognostic value of blood fibrinogen in predicting death risk in acute or exacerbated states of chronic HF. One recent study included a total of 554 HF patients from the Medical Information Mart for Intensive Care III (MIMIC III) database, and 90-day mortality was defined as the primary outcome^13^. They found that in the unadjusted Cox model, the 90-day mortality hazard ratio (HR) with 95% confidence intervals of the high fibrinogen level was 3.33 (95% CI 2.15–5.15) compared with the low fibrinogen level (< 284 mg/dL). The HR of the high fibrinogen level for 90-day mortality in multivariable Cox models changed slightly. They concluded that high fibrinogen levels (≥ 284 mg/dL) independently predict mortality in critically ill patients with acute exacerbation of chronic HF^13^. In contrast, our conclusion did not support the close correlation between elevated fibrinogen levels and a high risk for all-cause mortality, even though we observed a significant turning point at 2.4 g/L for blood fibrinogen. This difference may come from the fact that our outcome variable was all-cause mortality rather than disease-specific mortality. In another prospective cohort study of 1773 men aged 42–61 years who were free of HF or cardiac arrhythmias and whose plasma fibrinogen was measured at baseline, fibrinogen was positively and independently associated with the risk of sudden cardiac death during a 22-year follow-up^12^; however, those individuals mainly came from non-HF populations with younger ages from 42 to 61 years old, so their results are not comparable with those of our study. Moreover, our study data mainly came from patients discharged from the cardiology department after treatment, but their data came from patients in various ICUs (MIMIC III). Therefore, the difference in population selection may be an important reason for the difference in conclusions. Importantly, consistent with our findings, Chin et al.^14^ analyzed 120 patients with chronic and stable HF and did not observe that elevated fibrinogen levels were associated with a high risk for all-cause mortality. Similarly, our HF subjects were included in this analysis after receiving standard treatment for HF with a relatively stable condition. The only difference is that the follow-up time for our study was 6 months, while their follow-up time was 2 years.

The major advantage of our study was that a large sample of HF patients were enrolled in the study population from elderly patients admitted to a hospital in Sichuan, China, where there were no losses during the follow-up of 6 months and clinical variables were allowed for adequate adjustment. It is also the first exploration showing an inverted U-shaped association between blood fibrinogen and risk for readmission within 6 months in a Chinese population with HF, which provided more clinical evidence for previous controversial studies concerning HF prognosis. This may also provide a new candidate biomarker for the prognosis of HF patients in clinical practice that “blood fibrinogen = 2.4 g/L” among these patients had the highest risk for readmission within 6 months. Naturally, some limitations should be noted in this study. First, we could not control for the effects of unmeasured or unknown confounders for our findings, even though traditional CVD risk factors were controlled for. Second, fibrinogen in our study was only measured once at admission. Additional measurements of fibrinogen levels at discharge and/or follow-up may reliably determine the relationship of fibrinogen with prognosis. Third, the dataset was collected from a single center, so models developed using the data may not be generalizable. Fourth, the dataset did not offer time series data throughout the hospitalization period. For example, administration times for medications are not available. Thus, drug variables were not included in our analysis. Finally, we cannot clearly explain why there was no association between blood fibrinogen and all-cause mortality risk in the Chinese population, which is contrary to the results in previous investigations with the American population^13^. It is also unknown whether there are racial differences in this relationship. This should encourage more similar research to provide more evidence for our conclusion in different races.

## Conclusion

In summary, our findings suggested a curvilinear association (inverted U-shaped association) between blood fibrinogen and rehospitalization risk. The “blood fibrinogen = 2.4 g/L” among these patients had the highest risk for readmission within 6 months. This might also provide new evidence and perspective on the prognosis of blood fibrinogen for HF patients in clinical practice. certainly, more clinical evidence were needed to validate our conclusions.

## Data Availability

Our analysis data were obtained from an integrating electronic healthcare records and external outcome data (https://physionet.org/content/heart-failure-zigong/1.2/).
